# Influence of Personal Factors on Sound Perception and Overall Experience in Urban Green Areas. A Case Study of a Cycling Path Highly Exposed to Road Traffic Noise

**DOI:** 10.3390/ijerph15061118

**Published:** 2018-05-30

**Authors:** Francesco Aletta, Timothy Van Renterghem, Dick Botteldooren

**Affiliations:** Department of Information Technology, Ghent University, 9000 Ghent, Belgium; timothy.vanrenterghem@ugent.be (T.V.R.); dick.botteldooren@ugent.be (D.B.)

**Keywords:** urban green areas, soundscape, noise perception, noise annoyance

## Abstract

In contemporary urban design, green public areas play a vital role. They have great societal value, but if exposed to undue environmental noise their restorative potential might be compromised. On the other hand, research has shown that the presence of greenery can moderate noise annoyance in areas with high sound levels, while personal factors are expected to play an important role too. A cycling path bordered by vegetation, but highly exposed to road traffic noise, was here considered as a case study. A sound perception survey was submitted to participants on site and they were subsequently sorted into groups according to their noise sensitivity, visual attention and attitude towards greenery. The aim of this study was testing whether these three personal factors could affect their noise perception and overall experience of the place. Results showed that people highly sensitive to noise and more sceptical towards greenery’s potential as an environmental moderator reported worse soundscape quality, while visually attentive people reported better quality. These three personal factors were found to be statistically independent. This study shows that several person-related factors impact the assessment of the sound environment in green areas. Although the majority of the respondents benefit from the presence of visual green, policy-makers and planners should be aware that for a significant subset of the population, it should be accompanied by a tranquil soundscape to be fully appreciated.

## 1. Introduction

Quiet areas have been at the centre of the discourse on Environmental Noise in Europe for many years now. In 2002, the European Parliament and the Council issued the Environmental Noise Directive (END), which is the reference legal framework for Members States of the European Union for the “assessment and management of environmental noise” [1]. According to the END, each country should identify “quiet areas” and protect them from undue environmental noise. Since they were left to each country’s responsibility, the criteria for defining such areas are still a topic for debate and a lot of research efforts went in this direction over the past 15 years [2,3,4,5,6,7,8,9]. In 2014, the European Environment Agency (EEA) also tried to shed some light on this topic by publishing a “Good practice guide on quiet areas” [10]. In this policy document, the EEA suggests that (a combination of) four different methods could be used for the identification and management of quiet areas; these are: (1) noise mapping by modelling and calculations; (2) actual measurements of sound-pressure levels in situ; (3) evaluation of user/visitor experiences (i.e., the soundscape approach); and (4) expert assessments. Thus, actual noise levels are only one of the components to take into account. The EEA document also acknowledges for the first time the importance of the “soundscape approach”, which is related to how people perceive the acoustic environment [11].

It is reasonable to expect that, within urban agglomerations, *quiet* areas would tend to coincide with green areas, because they are typically associated with tranquillity and restorativeness [12,13]. The soundscape of green areas has been thoroughly investigated, for they offer a restorative potential and an opportunity for individuals (and groups) to retreat from everyday city noise, improving overall health and well-being [14,15,16]. Perceived quality of green areas and in particular soft connections may also stimulate people to walk and cycle, resulting in an additional benefit for health [17].

When highly exposed to environmental noise, the restorative potential of green areas might be compromised [18,19,20]. Notwithstanding, considering this issue from the opposite perspective, greenery features might also be seen as an important moderator of noise perception. Indeed, previous studies have shown that in complex environments, noise perception depends on a number of individual and non-acoustic factors, such as personal beliefs and preconceptions or vision-related elements [21,22,23,24,25].

In particular, Van Renterghem [26] reviewed a number of studies on the potentially positive effect of greenery features (and vegetation in general) on the perception of environmental noise. His review outlined three main mechanisms in which vegetation can possibly play a role as soundscape moderator; namely: visibility of the sound source, the mere presence of vegetation (either directly visible or accessibly nearby), and vegetation acting as a source of natural sounds (e.g., birdsong, wind-induced vegetation sounds, water features, etc.). In addition, Van Renterghem pointed out that personal characteristics might also be important factors in the interaction between vegetation and the perception of the sound environment (i.e., the soundscape). Therefore, this study aims to consider a number of such personal factors, using a case study of a cycling path in a green area highly exposed to road traffic noise.

Noise sensitivity has been recognised as a stable personal factor associated with noise annoyance at home since many years [27]. Although some researchers have criticised the significance of this factor, recent scientific evidence suggests it is associated with altered sound feature encoding and attenuated discrimination of sound noisiness in the auditory cortex [28]. This is consistent with earlier observations that noise sensitivity is at least partly inherited [29]. Therefore, noise sensitivity is included as a first personal factor in this study. The second personal factor included in this study is related to the interaction between the perception of the visual environment and the auditory environment. It was recently shown that in laboratory experiments, a distinction could be made between people that are easily visually distracted and those that have a stronger auditive focus. It was also shown that this classification influences the effect of visual context on evaluation of the sonic environment and the strength of the contribution of the sonic environment to the overall appreciation of an environment [30]. The third factor considered in this study is related to personal expectations and connections to “greenery” as an environmental mediator; that is a (positive) preconception people might have about the potential of natural features to compensate for negative effects of noise and air pollution [12,31].

Therefore, while acknowledging the potentially detrimental health effects that environmental noise can have on communities [32,33], this study aimed at exploring the three abovementioned personal factors related to noise perception in the public space. More specifically, a field survey was carried out and the differences in terms of overall experience of the place and perception of the sound environment were explored between groups of individuals sorted according to: (1) self-reported noise sensitivity; (2) self-reported visual distractibility; and (3) expectation about greenery’s potential to reduce air and noise pollution. This approach keeps together both the individual-related auditory and visual components of the experience of green areas, as well as another component related to individuals’ expectation and preconception about vegetation.

## 2. Methods and Materials

The following sub-sections briefly describe the investigated area and the procedures for data collection. These included both subjective data, i.e., the individual responses from the field survey and objective data, i.e., noise level measurements. The latter were only used to characterise the sound environment of the place, while the focus of the study is on the sound perception.

### 2.1. Case Study

**Figure 1:**
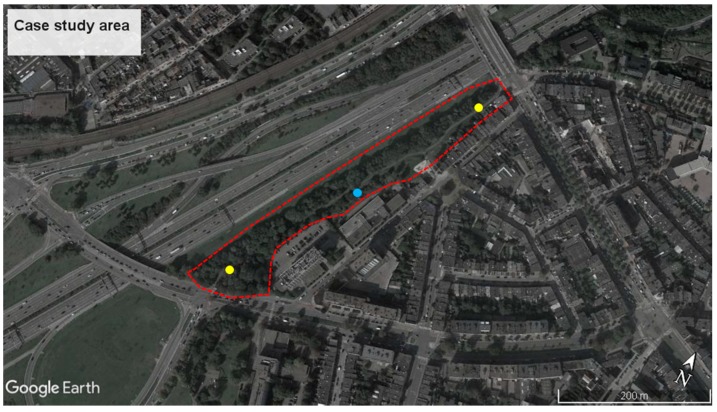
The investigated cycling path goes across the urban green area inside the red dashed line, bordering the ring road. The yellow dots represent the survey positions, at the end of the cycling path; the blue dot represents the microphone position.

The case study is located near a cycling path on an embankment, directly bordering a segment of the Antwerp Ring road (between Stenenbrug and Lippenslaan, see Figure 1). A 20-m deep vegetation belt with trees is present between the ring road and the cycling path. Also at the other side of the cycling path, trees are present. Although the total amount of biomass is rather limited, the tall trees give a strong impression of being “immersed” in green. During the survey, the vegetation was in full leaf. Passers-by are exposed to high noise levels due to the always intense road traffic on the ring road, which has 8/9 lanes near the zone under study.

### 2.2. Onsite Survey

There is still no unique procedure to gather data about the individuals’ soundscape experiences [34]. However, several protocols have been proposed over the years, and the sound-related items of the questionnaire used in this study are derived from some of those.

The questionnaire was organised into sections which covered a set of different topics (besides sound perception): (a) overall experience; (b) soundscape appraisal; (c) perceived loudness; (d) noise sensitivity; (e) visual distractibility; and (f) expected benefit of greenery. The item of category (a) relates to an overall assessment of the experience of using the cycling path [35,36]. The items of categories (b) and (c) are retrieved from soundscape literature and cover the perceived affective quality of the acoustic environment, using a set of soundscape dimensions [37,38] and the perceived loudness of the acoustic environment as a whole [20,39]. Category (d) consists of a reduced Dutch version of the Weinstein Noise Sensitivity Scale (WNSS) [40], as previous research has shown that a limited number of items can still accurately define profiles of users’ noise sensitivity [41]. The questions of category (e) relate to the individuals’ potential of being distracted by visual elements over auditory elements [25,30]. Eventually, the questions of category (f) refer to personal expectations towards greenery’s potential of mitigating noise from road traffic and improving air quality. The reason for including also an item about air quality in this category was trying to cover a broad attitude towards vegetation [12].

The questionnaire was administered by two research students at the cycling path, using an electronic form showed to volunteers through a smartphone, after seeking informed consent. Participants who successfully completed the questionnaire were given a small gadget provided by the City of Antwerp, as a token of appreciation for volunteering in the survey. Table 1 summarizes the questionnaire and the extremes of the scales participants could use. 

**Figure 2:**
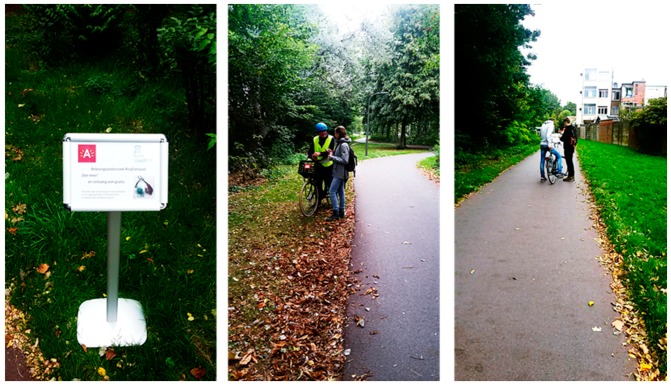
The panel on site announcing and advertising the survey, and photographs of the two research students approaching passers-by.

Data collection took place during five working days in September 2017 (from Monday the 4th to Friday the 8th), between 10 a.m. and 6 p.m. The research students worked simultaneously at the two ends of the path (Figure 1). They only approached passers-by leaving the path, while disregarding those entering the path (Figure 2). This is because the survey was meant to address people who had “just experienced” the path and could recall it clearly in their short-term memory.

During the week of observation, 181 valid responses to the survey were gathered. The research students informally reported that, considering all the people they approached, approximately one out of four decided to participate. Apart from the questions reported in Table 1, some basic demographics and general information were also collected: gender (M = 56%; F = 44%), age (M_age_ = 46.8; SD_age_ = 15.5), area of residence (less than 10 min away = 26%; in town = 35%; out of town = 39%). A further question about frequency and type of use was also asked, as shown in Table 2. It can be observed that most interviewees were cyclist (93%), using the path on a daily basis, as part of a trip to different destinations.

### 2.3. Road Traffic and Noise Levels’ Data Collection

**Figure 3:**
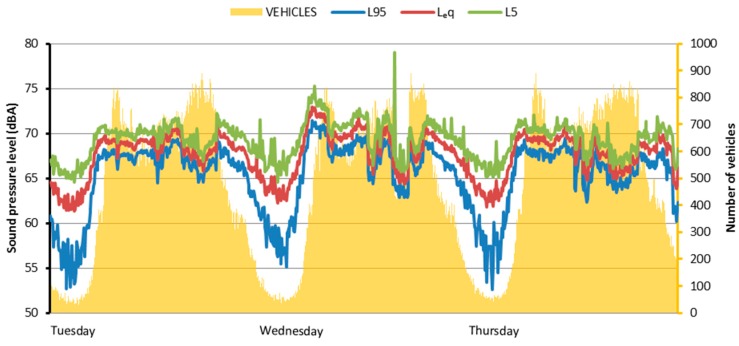
Noise level indicators and number of road vehicles (on 5-min intervals) during 3 days of the survey period. L_eq_: Equivalent sound pressure levels.

A semi-permanent type-1 microphone setup was installed on site, approximately halfway along the cycling path (Figure 1). Data from this microphone position accurately reflects the sound levels experienced by participants at the moment of the survey. It was measured that during the survey period equivalent sound pressure levels along the cycling path are near 70 dBA during the daytime. Equivalent sound levels (L_eq_), as well the 5th and 95th percentiles (L_5_ and L_95_) were calculated on a five-minute basis and are presented in Figure 3.

In order to better characterize the sound environment of the place, traffic data for the corresponding section of the Ring road were also sought from the Flemish Agency for Traffic (Vlaams Verkeerscentrum). Traffic data were provided as number of vehicles per minute crossing the section, with the vehicles sorted into: cars (<4.9 m), vans (5.0–6.9 m), trucks (7.0–11.9 m), and articulated trucks (>12.0 m). For the sake of comparison with noise level data, vehicle counts were summed across categories and aggregated on a five-minute basis as well.

**Figure 4:**
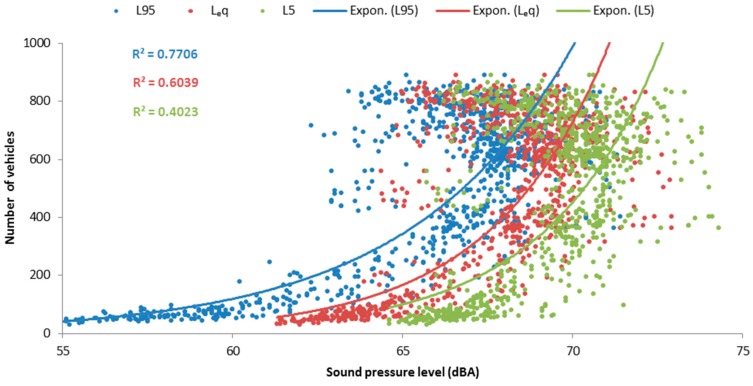
Exponential associations between sound levels and number of vehicles (on 5-min intervals).

Figure 3 shows an example of three days of monitoring during the survey period, representing both sound levels and vehicle counts. The correspondence between the daily patterns of the two datasets is clearly visible. Traffic saturation on the Ring Road is also noticeable: noise levels decrease during the intervals with highest traffic intensity, due to the consequent reduced speed of the vehicles. Furthermore, in Figure 4 it is possible to observe the exponential associations between the equivalent and statistical sound levels along the cycling path and the number of vehicles in transit on the Ring’s section. Going from the background levels (L_95_) to the peak levels (L_5_) the association with the number of vehicles becomes weaker. However, the number of vehicles accounts for more than 77% of variability in L_95_ values. Therefore, vehicles’ count and noise levels data confirm that the sound environment of the investigated green area is dominated by road traffic noise [42].

## 3. Results

Within the framework of this study, the questions in categories a–c (see Table 1) will be considered as “target” variables, while the questions in categories d–f (see Table 1) will be processed to define possible personal factors-related variables moderating the perception of the acoustic environment and overall experience on the cycling path. The three following sub-sections address the effects of the three main personal factors stated in the Introduction; i.e.: (1) self-reported noise sensitivity; (2) self-reported visual distractibility; and (3) expectation about greenery’s potential to reduce air and noise pollution. For each of these aspects, a categorical variable is defined to sort the sample into different groups.

### 3.1. Effect of Noise Sensitivity

One of the hypotheses underlying this study is that there could be differences in terms of soundscape and overall environmental experience, depending on personal factors, such as self-reported noise sensitivity. For this purpose, the reduced Dutch version of the standardized Weinstein Noise Sensitivity Scale (WNSS) was submitted to participants to assess their sensitivity profile (category d, in Table 1). For each of the ten statements (items), participants had to express a level of agreement on a five-point Likert scale ranging from “do not agree at all” (1) to “agree totally” (5). The last three items were “flipped” to match the direction of the others (i.e., higher scores imply higher sensitivity to noise).

For each participant, the arithmetic average of the ten items was calculated to derive a single “Noise Sensitivity Scale” (NSS) score. Participants were then sorted into two groups: (1) Low Noise Sensitivity, if the NSS score was lower than 3; (2) High Noise Sensitivity, if the NSS score was higher than 3. The two groups were then considered as levels of the categorical “NSS” variable.

A set of independent-samples *t*-tests was then run to determine if the scores of the survey items were different between the Low Noise Sensitivity (*n* = 45) and High Noise Sensitivity (*n* = 136) groups, as reported in Table 3.

**Figure 5:**
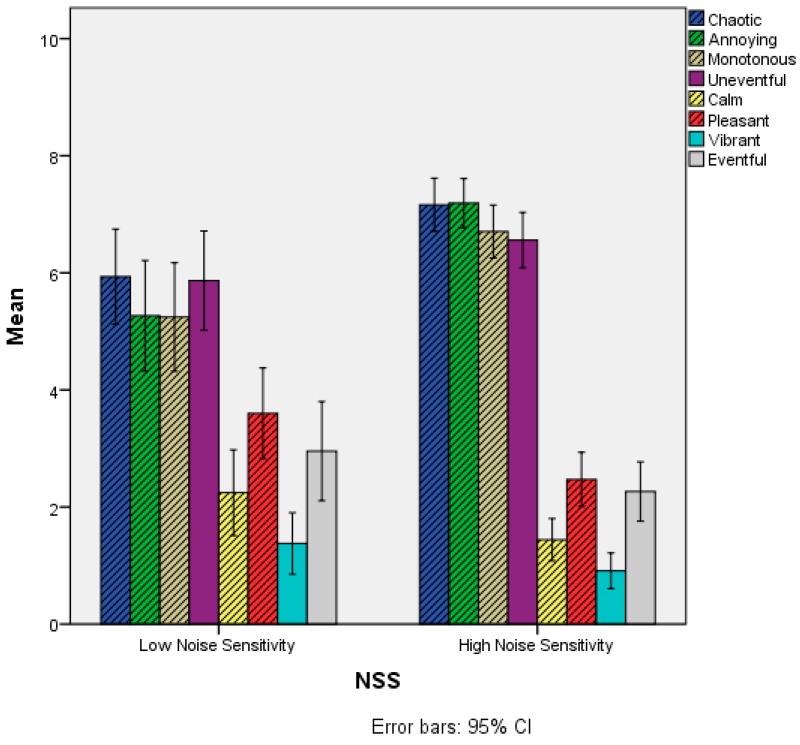
Mean scores of the Soundscape appraisal items as a function of the NSS variable. Grey-shaded bars refer to items where statistically significant differences were observed between the two groups. NSS: Noise Sensitivity Scale.

Regarding the Soundscape attributes, statistically significant differences emerged for the items Chaotic, Annoying, Monotonous, Calm and Pleasant. For the “positive” attributes (i.e., Calm and Pleasant), the scores of the Low Noise Sensitivity group were statistically significantly higher than the High Noise Sensitivity group, while for the “negative” items (i.e., Chaotic, Annoying and Monotonous), the scores of the High Noise Sensitivity group were statistically significantly higher than the Low Noise Sensitivity group, as reported in Figure 5 and Table 3.

**Figure 6:**
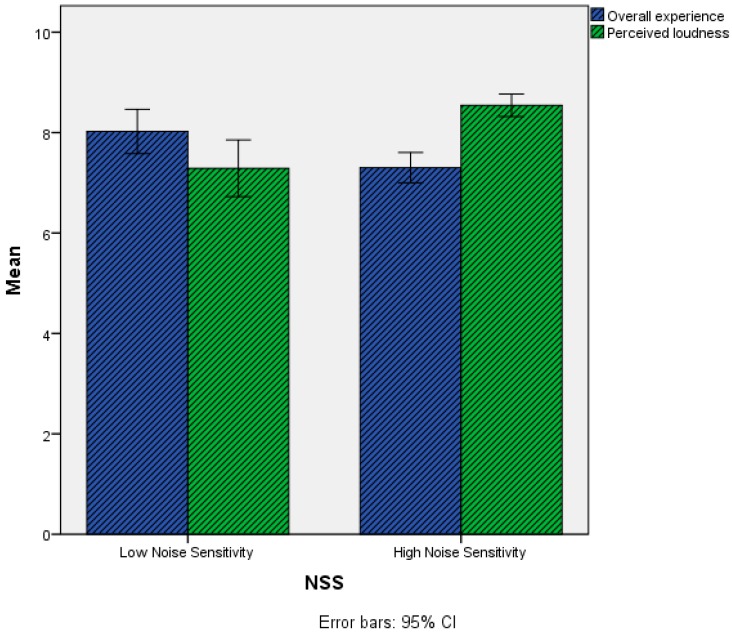
Mean scores of the Perceived loudness and Overall experience items as a function of the NSS variable. Grey-shaded bars refer to items where statistically significant differences were observed between the two groups.

For the Perceived loudness item the Low Noise Sensitivity (M = 7.29, SD = 1.88) group had statistically significantly lower scores than the High Noise Sensitivity group (M = 8.54, SD = 1.32). Contrariwise, for the Overall experience item the Low Noise Sensitivity (M = 8.02, SD = 1.47) group had statistically significantly higher scores than the High Noise Sensitivity group (M = 7.30, SD = 1.79). Mean scores for these two items are reported in Figure 6 and Table 3.

### 3.2. Effect of Visual Distractibility

In order to define a “Visual Distractibility” (VDT) variable, a *k*-means cluster analysis was performed on the scores of the three corresponding items of the questionnaire (Category e, in Table 1), forcing the algorithm into a two-cluster solution, since a convergence was achieved due to no or small change in cluster centres after less than ten iterations of the clustering algorithm (SPSS IBM v.22, SPSS Inc., Chicago, IL, USA). The process for defining the VDT variable is not the same as per the NSS variable, because in this case there are no standardized measures. Therefore, a clustering approach seemed the most suitable to offer insights into the structure of the interviewed sample [25].

Subsequently, the mean scores of the three VDT items were analysed as a function of cluster membership. Considering the positive direction of the three items, a high level of agreement can be seen as a higher individuals’ potential of being distracted by visual element over auditory elements. It was observed that the three items have always higher scores (even if mean differences change) for cluster 1 than for cluster 2; thus the two clusters were interpreted as: “High visual distractibility” (1) and “Low visual distractibility” (2). These were then considered as categorical levels of the “Visual distractibility” (VDT) variable.

A set of independent-samples *t*-tests was then run to determine if the scores of the survey items were different between the High visual attention (*n* = 94) and Low visual attention (*n* = 87) groups, as reported in Table 4.

**Figure 7:**
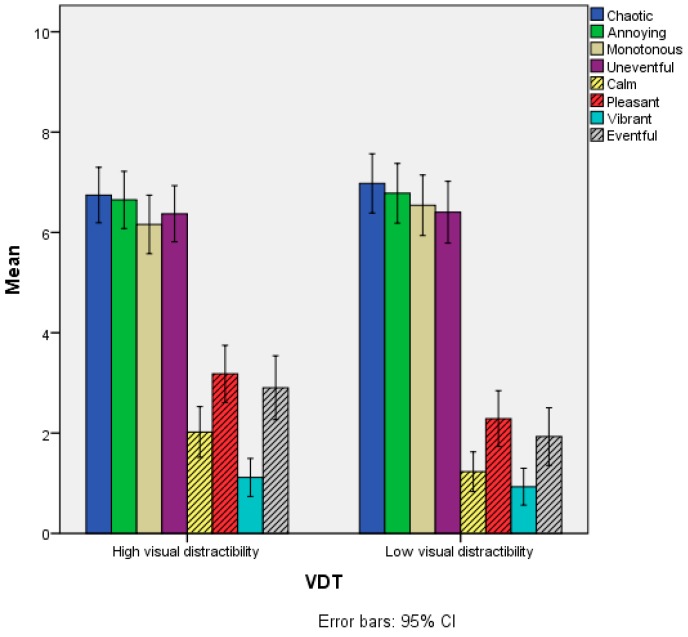
Mean scores of the Soundscape appraisal items as a function of the VDT variable. Grey-shaded bars refer to items where statistically significant differences were observed between the two groups.

Regarding the Soundscape attributes, statistically significant differences emerged only for the items Calm, Pleasant and Eventful. The scores of the High visual distractibility group were statistically significantly higher than the Low visual distractibility group, as reported in Figure 7 and Table 4.

**Figure 8:**
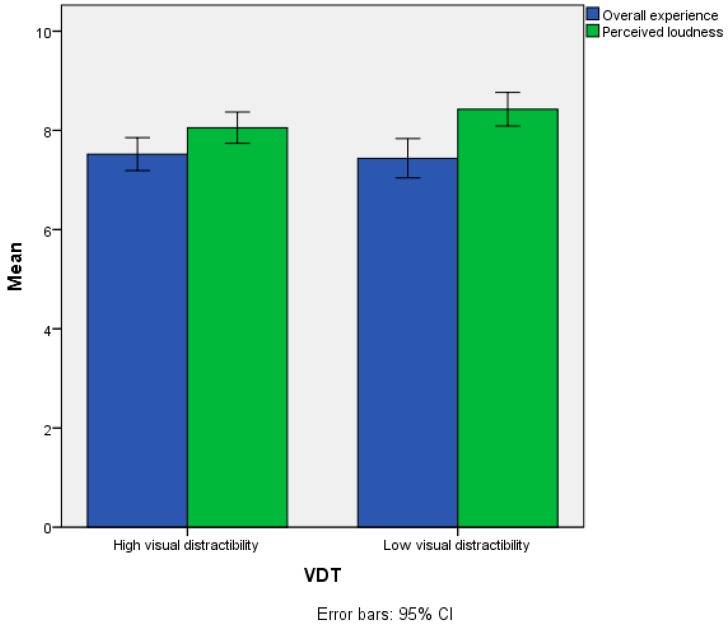
Mean scores of the Perceived loudness and Overall experience items as a function of the VDT variable.

For the Perceived loudness and Overall experience, no statistically significant differences (*p* > 0.05) were observed between the two VDT groups. Mean scores for these two items are reported in Figure 8 and Table 4.

### 3.3. Effect of Expected Benefit of Greenery

Similarly to what has been done for the VDT variable, a *k*-means cluster analysis was performed in order to define an “Expected Benefit of Greenery” (EBG) variable, using the scores of the three corresponding items of the questionnaire (Category f, in Table 1). The algorithm was forced into a two-cluster solution, since a convergence was achieved due to no or small change in cluster centres after only four iterations of the clustering algorithm (SPSS IBM v.22, SPSS Inc., Chicago, IL, USA).

Subsequently, the mean scores of the three ATG items were analysed as a function of cluster membership. Considering the positive direction of the three items, a high level of agreement can be seen as a positive attitude towards greenery in reducing the noise coming from the Ring road and improving the air quality. It was observed that the three items have always higher scores (even if to a different extent) for cluster 1 than for cluster 2; thus the two clusters were interpreted as: “Positive” (1) and “Sceptical” (2) about the benefit of greenery. These were then considered as categorical levels of the “Expected Benefit of Greenery” (EBG) variable.

A set of independent-samples *t*-tests was then run to determine if the scores of the survey items were different between the Positive (*n* = 114) and Sceptical (*n* = 67) groups, as reported in Table 5.

**Figure 9:**
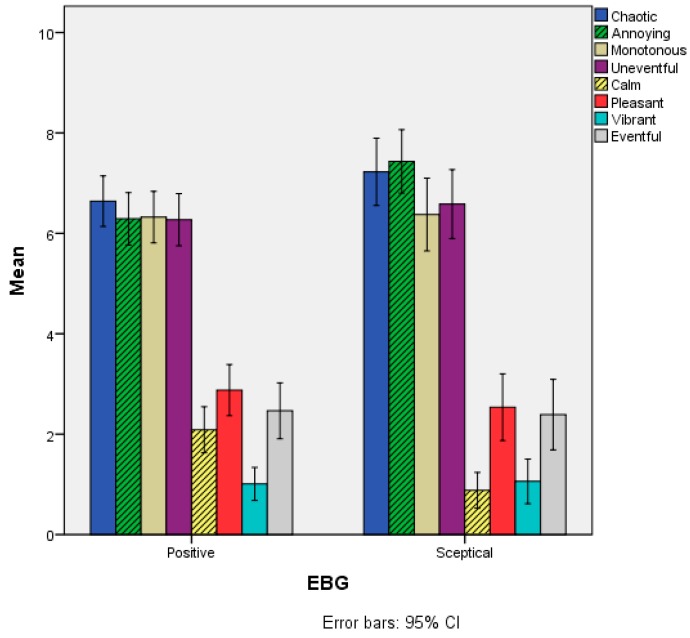
Mean scores of the Soundscape appraisal items as a function of the EBG variable. Grey-shaded bars refer to items where statistically significant differences were observed between the two groups.

Regarding the Soundscape attributes, statistically significant differences emerged only for the items Annoying and Calm. For Annoying, the scores of the Positive group (M = 6.29, SD = 2.81) were lower than the Sceptical group (M = 7.43, SD = 2.59). Conversely, for the Calm item, the scores of the Positive group (M = 2.09, SD = 2.47) were higher than the Sceptical group (M = 0.88, SD = 1.46), as reported in Figure 9. No statistically significant differences (*p* > 0.05) were observed between the two EBG groups for the other Soundscape appraisal items.

**Figure 10:**
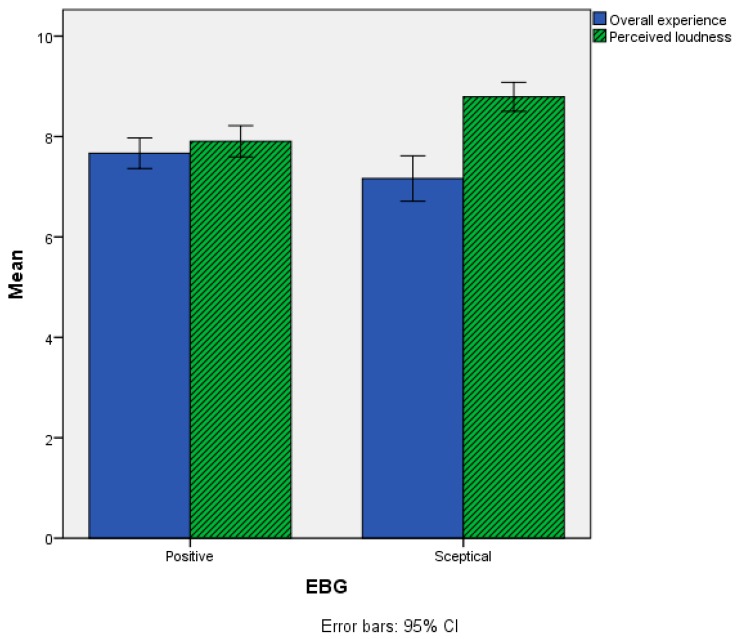
Mean scores of the Perceived loudness and Overall experience items as a function of the EBG variable. Grey-shaded bars refer to items where statistically significant differences were observed between the two groups.

For the Perceived loudness item the Positive (M = 7.90, SD = 1.68) group had statistically significantly lower scores than the Sceptical group (M = 8.79, SD = 1.19), but the difference in terms of Overall experience was not statistically significant between the EBG groups, as reported in Figure 10 and Table 5.

### 3.4. Relationship Between the Personal Variables

**Figure 11:**
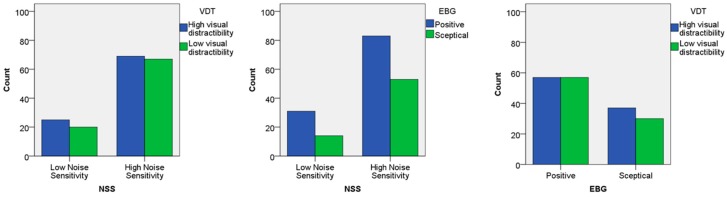
Bar charts representing the number of participants, sorted according to the groups of the three person-related variables (NSS*VDT, NSS*EBG, and EBG*VDT).

The assumption underlying the analysis so far was that the three variables (NSS, VDT and EBG) identified in this study to describe participants’ personal factors were independent. In order to statistically confirm this hypothesis, a set of three tests of association were conducted between the groups of the three variables, pairwise (i.e., NSS*VDT, NSS*EBG and EBG*VDT). The first chi-square test of independence was conducted between the NSS and VDT groups. All expected cell frequencies were greater than five. There was no statistically significant association between NSS and VDT groups, χ^2^(1) = 0.315, *p* = 0.575. The association was very small, Cramer’s V = 0.042. Similarly, the second chi-square test of independence was conducted between the NSS and EBG groups. All expected cell frequencies were greater than five. There was no statistically significant association between NSS and EBG groups, χ^2^(1) = 0.896, *p* = 0.344. The association was small, Cramer’s V = 0.070. Finally, the third chi-square test of independence was conducted between the EBG and VDT groups. All expected cell frequencies were greater than five. There was no statistically significant association between EBG and VDT groups, χ^2^(1) = 0.461, *p* = 0.497. The association, also in this case, was very small, Cramer’s V = 0.050. Figure 11 indeed shows no particular pattern in the distributions of participants across the groups of the three variables. Therefore, it was possible to assume that the three variables related to personal factors considered in this study are independent for the investigated sample.

## 4. Discussion

The hypothesis underlying this study was that in urban green spaces personal factors could affect the individual responses about the perception of the sound environment, both in terms of emotive appreciation (i.e., Soundscape appraisal) and magnitude of the auditory stimulus (i.e., Perceived loudness), as well as about the “holistic” perception of a place (i.e., Overall experience).

Within the framework of this study, Perceived loudness and Overall experience should be considered as independent “stand-alone” dimensions of the appreciation of the place. Indeed, while the Perceived loudness and Overall experience scores gathered on the cycling path resulted to be negatively related in a statistically significant way through a Pearson product-moment correlation analysis (*n* = 181, *p* = 0.007), their correlation was weak (*r* = −0.200) and Perceived loudness would only explain a limited amount (approx. 4%) of the variance in the Overall experience scores.

On the other hand, the Soundscape appraisal items should be regarded as part of a comprehensive perceived affective quality model, defined by two orthogonal components, annoying-pleasant and uneventful-eventful [37]. In this two-dimensional space (“circumplex” model) a soundscape that is: pleasant and eventful, is vibrant; pleasant and uneventful, is calm; annoying and eventful, is chaotic; annoying and uneventful, is monotonous [39].

**Figure 12:**
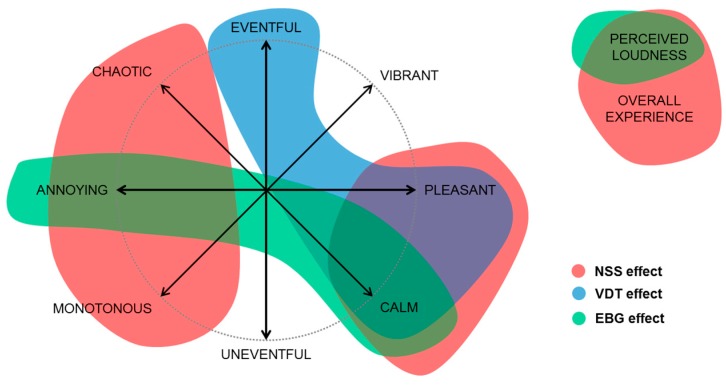
Schematic representation of the effects of personal factors (NSS, VDT and EBG variables) on Soundscape appraisal (depicted according to the “circumplex” model [37]), Perceived loudness and Overall experience.

Figure 12 represents the Soundscape appraisal model and the Perceived loudness and Overall experience items, and it shows where the three categories of personal factors (i.e., noise sensitivity, visual attention and attitude towards greenery) had significant effects on individuals’ responses. It can be seen that noise sensitivity (related to NSS) was influential in almost all dimensions of individuals’ responses. This is in line with previous literature, where it was reported that, when it comes to community response to environmental noise, “noise sensitivity” could be a better predictor than actual noise levels [43]. The lack of significance in the “eventfulness” dimension could be explained by the relatively stable (and loud) acoustic environment that interviewees were exposed to. That is, regardless of the noise sensitivity of the sample, differences in terms of eventfulness did not emerge because the physical acoustic environment did not offer enough temporal and/or spectral variability.

The “potential of being distracted by vision” factor (related to VDT) did not affect Perceived loudness and Overall experience. However, it did affect the “positive” region of the Soundscape appraisal model (i.e., vibrant-pleasant-calm). This suggests that, in the presence of visually distractive elements (like it could be the case for green features), visually-distractible people might tend to overestimate the positive dimensions of soundscapes, even when the acoustic environment is objectively (very) loud.

Finally, the expectation that greenery has the potential to reduce noise and improve air quality (related to ATG) only affected specific dimensions of sound perception, namely those related to the perceived loudness, the annoyance and the calmness. These findings also confirm what has been reported in literature. The “preference” for greenery plays an important role [44] and the restorative potential of green features is a dominant mechanism for noise annoyance [26]. This effect might have been enhanced as the exposure levels are high in the current case study [26]. The visibility of (good quality) green elements can enhance sustained attention restoration and stress relief, moderating the negative outcomes of environmental noise, and promote the perceptual construction of desired outputs, such as tranquillity and quietness (related to the attribute Calm, in this study) [3]. Another possible driver for the positive effects found here is that the cycling path is characterized by a strong visual immersion in natural green. This contrasts largely with the grey and highly urbanized region in its direct neighbourhood.

## 5. Conclusions

In this study, the perception of the sound environment and overall experience of a cycling path highly exposed to road traffic noise was analysed by means of a social survey submitted to 181 passers-by during daytime, in a typical working week. Previous studies have indeed shown that sound perception is likely to be modulated by personal factors, and this is particularly true when sound environments are experienced in urban green areas. Considering the relatively high and constant sound levels at the cycling path (“fixed” factor), the personal factors analysed in this study were: (1) self-reported noise sensitivity; (2) self-reported visual attention; and (3) expectation about greenery’s potential to reduce air and noise pollution; these were explored through the definition of three corresponding categorical variables (NSS, VDT and EBG) to sort the participants’ sample into groups. When looking at differences between groups, the main conclusions of this study are:

- People being highly sensitive to noise tended to overestimate the “negative” dimensions of soundscape appraisal and the loudness of the acoustic environment, compared to the less sensitive group, resulting in a “worse” overall experience of the cycling path.
- Instead, people being more visually attentive tended to be shifted towards some “positive” dimensions of soundscape appraisal.
- People having a positive attitude towards greenery experienced the soundscape along the cycling path as being less annoying, calmer and less loud than the group having a sceptical attitude.

It is important to point out that other personal factors might be playing a role in how people appraise soundscapes in urban green areas, such as their socio-economic status, or the type of area (e.g., residential, commercial, etc.) where the urban green is located [45]. Such factors were not controlled for in this research, and they might be the foundation for further empirical studies. However, it should be noticed that this case study is related to an urban green area in the proximity of the Ring road of Antwerp, which is a very unique transportation infrastructure, already at the centre of an environmental (and political) debate [46]. This work should be seen within a broader context of providing support to the design and management practice for urban green networks [47].

Altogether, the results of this study show that person-related factors can make a big difference when the sound environment of green public spaces is assessed. While this knowledge seems to confirm that designing green(er) public spaces can improve noise perception for the majority of people, policy-makers and planners should also keep this in mind when performing noise-related social surveys: sufficiently stratified samples of participants are then needed.

## Figures and Tables

**Table 1:** Summary of the onsite questionnaire. The information about the questions’ categories was not revealed to participants.

Question Category	Question(s)	Scale (extremes)
(a) overall experience	How would you generally describe your experience today when using the path between Stenenbrug and Lippenslaan?	Very bad—Very good (0–10)
(b) soundscape appraisal (Overall, the acoustic environment you just experienced was…)	Eventful	Not at all—Completely (0–10)
	Vibrant	
	Pleasant	
	Calm	
	Uneventful	
	Monotonous	
	Annoying	
	Chaotic	
(c) perceived loudness	When cycling/walking along the path, I rate the loudness of the environmental noise from the Ring road as…	Very quiet—Extremely loud (0–10)
(d) noise sensitivity (Please, state to what extent you agree to each of the following statements...)	No one should complain when one listens to music for a while	Do not agree at all—Totally agree (1–5)
	I wake up quickly because of noise	
	I get bothered when my neighbours are noisy	
	I get used to most noises without much trouble	
	Sometimes noise makes me nervous	
	Music that I usually love bothers me when I am trying to focus	
	I find it difficult to relax in a noisy place	
	It does not matter what’s happening around me, I can always concentrate well	
	I get angry with people making noise preventing me to sleep or work	
	I am sensitive to noise	
(e) visual distractibility (Please, state to what extent you agree with each of the following statements...)	I close my eyes to carefully listen to music	Do not agree at all—Totally agree (1–5)
	During a phone conversation I’m easily distracted from images on screens (computer, TV...)	
	I get tired quickly in a busy area	
(f) expectation towards greenery (Please, state to what extent you agree to each of the following statements...)	The vegetation located towards the ring road is able to reduce the road traffic noise at the cycling path	Do not agree at all—Totally agree (1–5)
	The vegetation located towards the ring road is able to improve the air quality at the cycling path	
	Being present in a green environment is good for your health	

**Table 2:** Frequency and type of use of the path by the 181 interviewees.

Reason for Using the Path	How Often do You Use this Path?						
	(Almost) Daily	Several Times A Week	About Once A Week	Several Times A Month	About Once A Month	Less than Once A Month	Rarely or Never
For leisure (e.g., cycling as recreation, sport, relax, etc.)	17.7%	13.8%	12.2%	8.3%	3.3%	5.0%	39.8%
To go somewhere (e.g., work, school, shops, etc.)	51.9%	20.4%	5.0%	1.1%	0.6%	1.1%	19.9%

**Table 3:** Independent-samples *t*-tests for the Soundscape appraisal, Perceived loudness and Overall experience items between the NSS groups.

Items (a–c)	*t*-Test for Equality of Means						
	t	df	Sig. (2-Tailed)	Mean Difference	Std. Error Difference	95% Confidence Interval of the Difference	
						Lower	Upper
Chaotic	−2.658	179	0.009	−1.228	0.462	−2.141	−0.316
Annoying	−4.215	179	0.000	−1.925	0.457	−2.826	−1.023
Monotonous	−3.064	179	0.003	−1.461	0.477	−2.403	−0.520
Uneventful	−1.441	179	0.151	−0.692	0.480	−1.640	0.256
Calm	2.116	179	0.036	0.803	0.380	0.054	1.552
Pleasant	2.436	179	0.016	1.129	0.464	0.215	2.044
Vibrant	1.522	179	0.130	0.466	0.306	−0.138	1.070
Eventful	1.364	179	0.174	0.691	0.506	−0.309	1.690
Perceived loudness	−4.939	179	0.000	−1.255	0.254	−1.757	−0.754
Overall experience	2.446	179	0.015	0.721	0.295	0.139	1.302

**Table 4:** Independent-samples *t*-tests for the Soundscape appraisal, Perceived loudness and Overall experience items between the VDT groups.

Items (a–c)	*t*-Test for Equality of Means						
	t	df	Sig. (2-Tailed)	Mean Difference	Std. Error Difference	95% Confidence Interval of the Difference	
						Lower	Upper
Chaotic	−0.570	179	0.569	−0.232	0.407	−1.036	0.571
Annoying	−0.320	179	0.749	−0.133	0.414	−0.950	0.684
Monotonous	−0.901	179	0.369	−0.381	0.422	−1.214	0.453
Uneventful	−0.072	179	0.943	−0.030	0.418	−0.855	0.795
Calm	2.419	179	0.017	0.791	0.327	0.146	1.437
Pleasant	2.222	179	0.028	0.893	0.402	0.100	1.687
Vibrant	0.699	179	0.486	0.186	0.266	−0.339	0.711
Eventful	2.241	179	0.026	0.973	0.434	0.116	1.830
Perceived loudness	−1.599	179	0.112	−0.372	0.233	−0.831	0.087
Overall experience	0.326	179	0.745	0.084	0.259	−0.427	0.596

**Table 5:** Independent-samples *t*-tests for the Soundscape appraisal, Perceived loudness and Overall experience items between the EBG groups. EBG: Expected Benefit of Greenery.

Items (a–c)	*t*-Test for Equality of Means						
	t	df	Sig. (2-Tailed)	Mean Difference	Std. Error Difference	95% Confidence Interval of the Difference	
						Lower	Upper
Chaotic	−1.391	179	0.166	−.584	0.420	−1.412	0.244
Annoying	−2.723	179	0.007	−1.143	0.420	−1.972	−0.315
Monotonous	−0.111	179	0.912	−0.049	0.438	−0.913	0.816
Uneventful	−0.718	179	0.474	−0.310	0.432	−1.162	0.542
Calm	3.637	179	0.000	1.207	0.332	0.552	1.862
Pleasant	0.807	179	0.421	0.340	0.421	−0.491	1.171
Vibrant	−0.185	179	0.854	−.051	0.276	−0.595	0.493
Eventful	0.169	179	0.866	0.077	0.456	−0.822	0.976
Perceived loudness	−3.805	179	0.000	−0.888	0.233	−1.348	−0.427
Overall experience	1.892	179	0.060	0.502	0.266	−0.021	1.026
